# Kidney function decline improves after lithium discontinuation

**DOI:** 10.1111/joim.20054

**Published:** 2025-01-20

**Authors:** Filip Fransson, Ursula Werneke, Louise Öhlund, P. Andreas Jonsson, Michael Ott

**Affiliations:** ^1^ Department of Public Health and Clinical Medicine Umeå University Umeå Sweden; ^2^ Department of Clinical Sciences, Psychiatry, Sunderby Research Unit Umeå University Umeå Sweden

**Keywords:** bipolar disorder, chronic kidney disease, lithium, renal function

## Abstract

**Background:**

Long‐term lithium treatment decreases kidney function. However, it remains unclear whether stopping lithium improves kidney function.

**Objectives:**

To study kidney function in patients who stopped and subsequently restarted lithium treatment.

**Methods:**

Mirror‐image design using data from the LiSIE retrospective cohort study. The mirror was set to when lithium was stopped with a 5‐year pre‐ and post‐mirror period. Adult patients with bipolar, schizoaffective disorder or unipolar depression, who had lithium ≥4.5 years in the pre‐mirror period, were included. Creatinine measurements were available from 1997 to 2017. The main outcome was the difference in mean annual change of the estimated glomerular filtration rate (eGFR) adjusted for sex, hypertension and diabetes mellitus.

**Results:**

A total of 168 participants (94 women, 74 men) were included. Mean annual eGFR change was −1.58 (−1.87 to −1.28) mL/min/1.73 m^2^/year before and −0.023 (−0.49 to +0.44) mL/min/1.73 m^2^/year after lithium discontinuation (*p* < 0.0001 for difference). The improvement was 0.77 (0.35–1.20) mL/min/173 m^2^/year in participants with eGFR >60 mL/min/1.73 m^2^, and 3.03 (2.15–3.92) mL/min/1.73 m^2^/year for participants with eGFR <30 mL/min/1.73 m^2^. The effect was persistent over the 5‐year post‐mirror study period. For participants restarting lithium, the mean annual eGFR change was −1.71 (−2.26 to −1.16) mL/min/1.73 m^2^/year, a setback compared to their lithium‐free post‐mirror period (*p* < 0.0001). We did not see any difference compared to the pre‐mirror period (*p* = 0.51).

**Conclusions:**

Stopping lithium slowed down mean eGFR decline. This effect was more pronounced in participants with lower eGFR at the time of lithium discontinuation. In participants who restarted lithium, the annual decline of eGFR reverted to pre‐lithium discontinuation levels.

## Graphical abstract

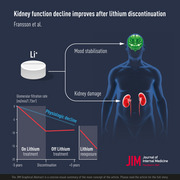

## Introduction

Lithium salts have been a mainstay in the treatment of mood disorders since the 1970s. Current guidelines support lithium as a first‐line drug for relapse prevention in bipolar disorder (BD) [1]. Lithium is thought to lower the risk of suicide and to be superior to other mood stabilizers [2]. Apart from being used as a maintenance treatment for BD, lithium is also used for the treatment of acute mania or as an augmentation therapy for unipolar depression. However, long‐term lithium treatment causes faster loss of kidney function [3]. Decreased kidney function is a strong risk factor for cardiovascular morbidity and mortality [4]. It may also increase the risk of lithium intoxication [5, 6]. Finally, kidney failure can ensue. Lithium‐associated glomerular damage (lithium nephropathy) has been at the forefront of clinicians’ minds since the early 1980s. This gave rise to the question of whether the benefits of lithium to mental health were bought at the expense of renal health [7]. Treatment strategies to address renal side effects of lithium remain lacking even today [8].

Intuitively, stopping lithium could be considered the most effective way to prevent the progression of lithium nephropathy. In our previous work, we have shown that decreasing glomerular filtration rate (GFR) accounts for 9% of lithium discontinuations [9]. However, it remains unclear whether stopping lithium improves kidney function. We searched PubMed for studies published between 1 January 2003 and 11 August 2024, using the search string ‘lithium AND kidney’ or ‘lithium AND renal’, without language restrictions. We also searched PubMed for systematic reviews using the same search strings without time restriction. We identified 1271 studies and 31 systematic reviews. These, we screened for data on the effect of lithium discontinuation on kidney function, reported either as GFR or creatinine clearance. We then complemented the search with additional references from the identified studies and systematic reviews. In total, we identified 10 studies of which 3 showed significant improvement and 7 no significant difference (Supporting Information Table A1) [10, 11, 12, 13, 14, 15, 16, 17, 18, 19]. Even a ‘point‐of‐no‐return’, beyond which kidney function irrevocably continues to decline after lithium discontinuation, has been suggested [20, 21].

The current work had three aims: To determine whether (a) estimated GFR (eGFR) changed after lithium discontinuation in participants who had been exposed to lithium long‐term, (b) there existed a ‘point‐of‐no‐return’ and (c) eGFR decline accelerated again if lithium was subsequently restarted.

## Methods

### Study design

The current study was a mirror‐image study examining eGFR within 5 years before and after lithium discontinuation using data from the LiSIE (lithium‐study into effects and side effects) research programme. LiSIE is a retrospective cohort study conducted in the Swedish regions of Norrbotten and Västerbotten. For the current study, only participants from the Norrbotten region were included since medical records from participants from the Västerbotten region were only partially accessible.

LiSIE procedures, including sampling and consent, have been described in detail previously [3]. In brief, LiSIE recruited adult individuals with BD, schizoaffective disorder (SZD) or depression with lithium treatment. Deceased individuals were included in accordance with the ethical approval granted. Living individuals were informed in writing about the nature of the LiSIE study and provided verbal informed consent. The consent was then documented in our research files, dated and signed by the research worker who obtained the consent. Consent procedures concluded by the end of 2012. The cohort was locked at this point; no new participants were included in the study thereafter. About 75% of the approached patients consented. Including deceased individuals, LiSIE covers 80% of the eligible individuals. For all included participants, available data from 1965 until 31 December 2017 were analysed. All procedures were approved by the Regional Ethics Review Board at Umeå University, Sweden (DNR 2010‐227‐31M, DNR 2011‐228‐32M, DNR 2014‐10‐32M and DNR 2018‐76‐32M). The funders of the study had no role in study design, data collection, data analysis, data interpretation or writing of the report.

### Participants

For this study, we included participants whose lithium treatment had been stopped between 1 January 1997 and 31 December 2013. Medical records were examined for termination of the prescription or lack of serum lithium concentrations. The cause and exact time of lithium discontinuation were manually validated independently by two researchers. We then selected participants with (a) a cumulative time on lithium of at least 4.5 years within the 5 years prior to discontinuation and (b) continuous lithium treatment during the 3 months preceding discontinuation. When there were several episodes of lithium discontinuation, we included the episode with the longest lithium‐free follow‐up. To enable a comparison of the two mirror periods under equal conditions, only participants with known kidney diseases expected to have a slow and continuous GFR decline pattern were included. Participants with kidney diseases where irregular or rapid GFR decline patterns could occur were excluded (in the ‘Method’ section of Supporting Information). We further excluded participants in whom, after manual validation, a diagnosis of schizophrenia or personality disorder was more likely than BD or SZD. Sex was defined according to legal sex stated in the medical records on 31 December 2017. Case notes were evaluated until 31 December 2017. The following comorbidities were recorded: diabetes mellitus, arterial hypertension, smoking, cardiovascular disease, concurrent renal diseases, lithium intoxications and prescriptions of renin‐angiotensin‐aldosterone‐inhibitors (criteria in the “Method” section of Supporting Information). Participants were, according to local practice, prescribed lithium sulphate twice daily. Some were prescribed differently, either lithium sulphate once daily or lithium citrate (in the ‘Method’ section of Supporting Information).

### Mirror periods

The mirror was set to the point when lithium was stopped. If lithium was tapered, the date of first dose reduction was used. The pre‐mirror period was set to 5 years. The post‐mirror period was also set to 5 years unless another event interfered. Such events included (a) the start of chronic renal replacement therapy, (b) death, (c) the end‐of‐study date 31 December 2017, (d) restarting lithium, defined as continuous treatment for longer than 90 days or (e) occurrence of other somatic diseases either affecting kidney function or leading to a loss of muscle mass impeding interpretation of creatinine‐based eGFR. If a participant was diagnosed with a somatic disease affecting creatinine interpretation already in the pre‐mirror period, data were only included up to the time of diagnosis, excluding all data from the post‐mirror period. For the analysis of eGFR changes after restarting lithium, the observation time was extended to a further minimum of 2 years after reinstatement, that is, an observation time of maximal 7 years in participants who had discontinued and then reinstated lithium (Supporting Information Fig. A1).

## Procedures

### Measurement of kidney function and estimation of GFR

Measurements of creatinine were available from 1 January 1997 to 31 December 2017 accessible through the central laboratory database, which included all measurements taken in public or private health care. All blood samples had been analysed using enzymatic methods. Samples from 2004 or later had been analysed by an assay calibrated to be traceable to isotope dilution mass spectrometry creatinine. Blood samples before 2004 had been analysed by an assay calibrated to the manufacturer's specifications at that time. These we converted before statistical analysis to make them comparable to the newer samples. We included all measurements from the pre‐ and post‐mirror period and the period after restarting lithium. We calculated eGFR from creatinine, age and sex with the Lund‐Malmö revised formula, validated in a Swedish population [22, 23]. Creatinine samples taken during episodes of acute kidney injury (AKI) were censored (criteria in the ‘Procedures’ section of Supporting Information).

### Outcome

The outcome was the difference in mean annual eGFR change in mL/min/1.73 m^2^ between the pre‐ and post‐mirror periods adjusted for sex, hypertension and diabetes.

### Statistical analysis

We first analysed the data descriptively. To test for differences in key parameters between the pre‐ and post‐mirror period or subgroups of different eGFR at discontinuation, we used a two‐tailed paired *t*‐test. Using all available creatinine measurements, the annual mean eGFR change was analysed as the dependent (outcome) variable, using a mixed‐effect model with random slopes and intercepts. Fixed effects were age, sex, years since discontinuation of lithium, hypertension and diabetes. All effects on the slope were assumed to be linear (full model design and syntax in the ‘Statistical analysis’ section of Supporting Information). We further conducted a subgroup analysis for different chronic kidney disease (CKD) stages. For this, we stratified participants in non‐mutually exclusive groups, according to eGFR at the time of lithium discontinuation: eGFR 0 to <30 (CKD stages G4–G5), 0 to <45 (CKD stage G3b to G5), 0 to <60 (CKD stages G3a–G5) or ≥60 mL/min/1.73 m^2^. We then repeated the analysis after the reinstatement of lithium. For comparison with older studies, we also analysed the data using linear regression (least squares). To test for differences between participants having improved or not improved after stopping lithium, we used chi‐square test and the Mann‐Whitney *U* test. To visualize the trend in kidney function changes over the whole study period, we calculated the mean annual eGFR from all creatinine measurements available per year per participant during the study period. Statistics were conducted with R version 4.30 [24] and SPSS version 25 (IBM).

### Choice of primary measure

As primary measure, we chose the comparison of annual eGFR change in terms of slopes. Using slopes yields better power in smaller populations with shorter follow‐up. Therefore, slopes can pick up significant eGFR changes when comparison of absolute eGFR values cannot. Annual eGFR changes in terms of slopes have been proposed as an adequate surrogate marker for kidney outcome in randomized clinical trials [25]. An upward slope change of 0.75 mL/min/1.73 m^2^/year over 3 years predicted a clinical benefit on CKD progression [25].

### Control for bias and missing data

In LiSIE, we controlled for selection bias with key parameters available in anonymized form. These included age, sex and, where applicable, maximum recorded concentrations of lithium and creatinine. In accordance with the ethics approval granted, we compared these parameters for consenting and non‐consenting participants. There were no significant differences between the two groups. The data were complete for included participants for the defined outcome.

## Results

**Fig. 1:**
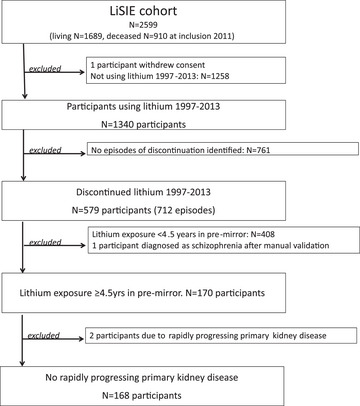
Participant selection.

Of 579 participants having discontinued lithium, we included 168 (94 females, 74 males) participants with sufficiently long exposure (Fig. 1).

Due to missing records, the exact historical lifetime lithium exposure could not be calculated for ten participants. Historical mean lithium levels could not be calculated for one participant. For 24 participants, smoking status was not reported. All other data were complete. The baseline characteristics of participants are shown in Table 1.

**Table 1:** Baseline characteristics at mirror.

**Age (years)**	
Mean (SD)	62 (14)
Minimum–maximum	24–91
**Sex, *N* (%)**	
Female	94 (56)
Male	74 (44)
**Psychiatric diagnosis, *N* (%)** ^a^	
Bipolar disorder type 1	81 (48)
Unspecified bipolar disorder	30 (18)
Bipolar disorder type 2	25 (15)
Unipolar depression	19 (11)
Schizoaffective disorder	13 (8)
**Diabetes mellitus**	
Number of participants (%)^b^	28 (17)
Duration in years, mean (SD)	11 (8)
**Hypertension**	
Number of participants (%)	72 (43)
Duration in years, mean (SD)	10 (10)
**Smoking** ^c^	
Number of participants (%)	63 (44)
**Cardiovascular disease**	
Number of participants (%)	38 (23)
**Reason for discontinuation, *N* (%)**	
Somatic reasons, all	99 (59)
*‐Whereof mainly due to renal cause* ^d^	56 (33)
*‐Whereof mainly due to low eGFR*	42 (25)
Lack of effect	24 (14)
Other psychiatric reasons^e^	15 (9)
Non‐adherence	14 (8)
Intoxication	11 (7)
Stopped due to palliative care	5 (3)
**Event ending study period, *N* (%)**	
End of mirror	71 (42)
Restarting lithium	48 (29)
Death	22 (13)
Severe disease or medical procedure^f^	19 (11)
End of study period	8 (5)
Chronic renal replacement therapy	0 (0)
**Lifetime exposure to lithium (years)** ^g^	
Mean (SD)	15 (10)
Minimum–maximum	5–43
**Historical lithium levels (mmol/L)** ^h^	
Mean (SD)	0.61 (0.13)
**History of lithium intoxication)** ^i^	
Number of participants (%)	15 (9)
**AKI at the time of lithium discontinuation**	
Number of participants (%)	23 (14)
**Last eGFR before discontinuation (mL/min/1.73 m^2^)**	
Mean (SD)	67 (23)
Minimum–maximum	8–127
≥60 (no CKD, CKD G1 or G2), *N* (%)	110 (65)
45–59 (CKD G3a), *N* (%)	27 (16)
30–44 (CKD G3b), *N* (%)	17 (10)
15–29 (CKD G4), *N* (%)	13 (8)
≤15 (CKD G5), *N* (%)	1 (1)

Abbreviations: *N*, number; IQR, interquartile range; SD, standard deviation; AKI, acute kidney injury; eGFR, estimated glomerular filtration rate.

^a^ Diagnosis at lithium initiation.

^b^ 1/28 participants had T1DM.

^c^ At any time since 1997. Data available for *N* = 144.

^d^ Decreasing kidney function, polyuria, polydipsia, nephrogenic diabetes insipidus.

^e^ Psychiatric side effects, fear of future side effects, or when the participant had discontinued without disclosing the cause.

^f^ See in the ‘RESULTS’ section of Supporting Information.

^g^ Data available for *N* = 158.

^h^ Since 1997, data available for *N* = 167.

^i^ ≥1.5 mmol/L at any time since 1997.

The pre‐mirror period included 833 patient‐years, median of 5.00 (interquartile range [IQR] 0.00) years/participant. The post‐mirror period included 502 patient‐years, median of 4.15 (IQR 4.46) years/participant. Due to AKI, 396 creatinine measurements in 64 episodes were censored. In total, 5011 measurements in 166 participants in the pre‐mirror period and 139 participants in the post‐mirror period were included in the final analysis. The number of measurements/year/participant differed between the pre‐ and post‐mirror periods (mean 4.52 (standard deviation [SD] 2.24) vs. mean 2.49 (SD 3.16), *p* < 0.0001). Treatment with RAAS (renin–angiotensin–aldosterone‐system) inhibitors was less prevalent during the pre‐mirror than the post‐mirror (mean 9% of days/year (SD 24) vs. mean 13% of days/year (SD 31), *p* = 0.0092). AKI episodes per year did not differ (Supporting Information Table A2).

In the mixed model analysis, the mean annual eGFR change before lithium discontinuation (pre‐mirror period) was −1.58 (95% confidence interval [CI]: −1.87 to −1.28) mL/min/1.73 m^2^/year. The mean annual eGFR change after lithium discontinuation (post‐mirror period) was −0.023 (95% CI: −0.49 to +0.44) mL/min/1.73 m^2^/year. The difference attributable to discontinuing lithium was therefore 1.55 (95% CI: 1.23 to 1.87) mL/min/1.73 m^2^/year, *p* < 0.0001. The effect attributable to hypertension calculated over the whole study period was −0.65 (95% CI: −1.05 to −0.26) mL/min/1.73 m^2^/year, *p* = 0.0011. The effect on eGFR attributable to diabetes was −0.66 (95% CI: −1.21 to −0.11) mL/min/1.73 m^2^/year, *p* = 0.019. The effect of sex was not statistically significant, 2.56 (95% CI: −4.53 to 9.68) mL/min/1.73 m^2^, *p* = 0.47.

**Fig. 2:**
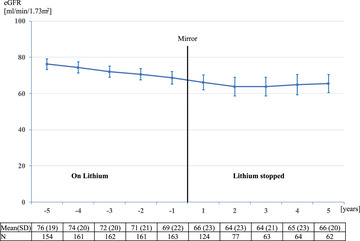
Mean annual estimated glomerular filtration rate. N = 168. Error bars: 95% confidence intervals. SD, standard deviation.

The effect of lithium discontinuation on eGFR was persistent over the 5 years of the post‐mirror period (Fig. 2, Fig. A2) and significant over all ranges of kidney function (Table 2). Participants with low eGFR at the point of lithium discontinuation had the largest effect (Table 2). Historical mean lithium levels differed between participants with eGFR <60 compared to eGFR ≥60 at discontinuation; 0.64 versus 0.59 mmol/L (*p* = 0.0081).

**Table 2:** The effect of lithium discontinuation in participants with low estimated glomerular filtration rate (eGFR) mL/min/1.73 m^2^/year.

	All	eGFR ≥60	eGFR <60	eGFR <45	eGFR <30
*N*	168	110	58	31	14
Difference in slope after discontinuation^a^	1.55	0.77	2.47	2.85	3.03
95% CI for difference	1.23–1.87	0.35–1.20	2.02–2.93	2.25–3.44	2.15–3.92
*p*	<0.0001	0.0003	<0.0001	<0.0001	<0.0001

*Note*: Groups are non‐mutually exclusive.

Abbreviations: CI, confidence interval; eGFR, estimated glomerular filtration rate.

^a^ Difference in annual change of eGFR between pre‐ and post‐mirror period. Positive values represent an improvement of the annual change of eGFR.

**Fig. 3:**
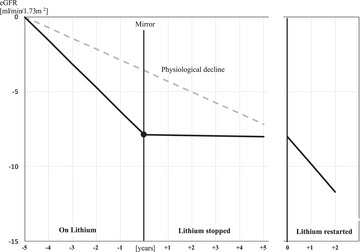
Model for annual change of estimated glomerular filtration rate (eGFR) during periods of lithium treatment, after stopping and after restarting lithium taking into consideration the effect of physiological aging according to Denic et al. [26].

In total, 48 participants reinstated lithium after a median of 0.51 (IQR 1.05) years of lithium discontinuation. Thereafter, data were included for a median of 2.81 (IQR 3.56) years with 732 creatinine samples available for analysis. Events ending the observation period after restarting lithium are presented in Supporting Information Table A3. The annual eGFR change after restarting lithium was −1.71 (CI 95% −2.26 to −1.16) mL/min/1.73 m^2^/year. The difference compared to the lithium‐free post‐discontinuation slope was −1.52 (CI 95% −2.08 to −0.96) mL/min/1.73 m^2^/year, *p* < 0.0001. The difference in annual change of eGFR to the pre‐mirror slope was non‐significant with −0.15 (CI 95% −0.61 to +0.30) mL/min/1.73 m^2^/year, *p* = 0.51. The findings are illustrated in a model for eGFR changes with or without lithium in comparison to the expected physiological changes [26] (Fig. 3).

In 88 participants, there was sufficient data to calculate the slopes of annual eGFR change before and after discontinuation with linear regression (Supporting Information Table A4). Slopes were negative in 72 (82%) participants before discontinuation (i.e., annual loss of eGFR) and in 66 (75%) participants thereafter. In 53 (60%) participants, the slope improved after discontinuation. In 19 (26%) participants, the slope even changed to positive (i.e., annual gain of eGFR). The last mean eGFR prior to lithium discontinuation did not differ between participants who changed from negative to positive slope and those who did not (58.82 (SD 20.34); 61.77 (SD 22.94); *p* = 0.69). In the 19 participants with eGFR <45, the slope improved in 15 (79%). Of these, five (33%) changed to a positive slope. In the 10 participants with eGFR <30, the slope improved in 9 (90%) cases. Of these, two (20%) changed to a positive slope. Annual decline of kidney function steeper than 2 mL/min/1.73 m^2^/year (*p* < 0.0001), low eGFR (<60 mL/min/1.73 m^2^) at discontinuation (*p* = 0.0059) and declining kidney function as reason for discontinuing lithium (*p* = 0.017) were predictors of eGFR slope improvement. Other relevant predictors did not reach the pre‐set significance level of *p* = 0.05; hypertension (*p* = 0.070), diabetes mellitus (*p* = 0.058) and lifetime lithium exposure ≥ = 10 years (*p* = 0.059) (Supporting Information Tables A5 and A6).

## Discussion

Lithium discontinuation slowed down the mean eGFR decline in the whole study group. This observation was consistent throughout the 5‐year observation period after lithium discontinuation. The effect was more pronounced in participants with lower eGFR at the time of lithium discontinuation. In participants who restarted lithium, the mean annual decline of eGFR reverted to that of pre‐lithium discontinuation levels. To our knowledge, this is the first study demonstrating a bi‐directional association between lithium treatment and kidney function, in that the decline of eGFR improves when lithium is discontinued and worsens again when lithium is reinstated.

In our literature review, we found 10 other studies exploring the effect of lithium discontinuation on kidney function, reported either as GFR or creatinine clearance (Supporting Information Table A1) [10, 11, 12, 13, 14, 15, 16, 17, 18, 19]. Two additional studies re‐analysed datasets from the previous 10 studies [27, 28]. Of all identified studies, three showed significant improvement, eight no significant difference after stopping lithium, and one report found kidney function decline to be significantly accelerated after discontinuation [27]. This report is an update on a previous study [15]. The original study did not find any difference when comparing eGFR over 4 years in participants with eGFR <45 in whom lithium was continued compared to participants in whom lithium was reduced or discontinued. The later follow‐up found a difference in favour of continuing lithium. Details on the statistical analysis and characteristics of participants were not reported, making comparisons to our results difficult.

One of the studies finding an improvement used a mirror‐image design. Like in our study, the difference in annual eGFR change was reported. The authors found a difference in slope of 2.0 mL/min/1.73 m^2^/year in favour of lithium discontinuation [13]. This is roughly in line with our findings. Participants with CKD (eGFR <60 mL/min/1.73 m^2^) at the start of the study were excluded. This makes it difficult to compare our findings in higher stages of CKD. Another study showing significant improvement in kidney function was a prospective lithium‐withdrawal study. It found GFR to improve with a mean follow‐up of only 3 months [10]. The third study showing significant improvement in kidney function used peak creatinine as a reference, which makes comparison with our study impossible [17].

Of the studies that did not detect a certain difference in kidney function, one was a prospective lithium‐withdrawal study with 13 participants finding no difference after two months [11]. Two other studies compared eGFR in participants who continued or discontinued lithium having developed CKD (eGFR <60) without finding any difference [14, 16]. In these studies, the change in annual eGFR loss before and after discontinuation was not reported. This made a possible relative improvement in the annual decline of kidney function difficult to detect.

The remaining studies either included few participants discontinuing lithium [18, 19] or had changes in laboratory methods during the study period limiting interpretation [12].

In summary, our literature review revealed that most studies had failed to detect a renal benefit of lithium discontinuation. All studies had small sample sizes of participants discontinuing lithium (*N* ≤ 46), making it difficult to reach statistical significance. Only one study was designed to detect improvement in the annual decline of kidney function. We did not find any study reporting on the renal effect of reinstating lithium.

Our finding of an additional annual decline in eGFR due to arterial hypertension and diabetes mellitus is in line with other studies in lithium‐treated participants, in which hypertension [3, 29] and diabetes mellitus [14, 30] significantly affected GFR. Smoking was common among participants in our study. Previous studies found comparable numbers [31]. Current and former smokers had increased odds of CKD compared with never smokers [32]. The effect of smoking on kidney function is complex as current smoking seems to lead to hyperfiltration and higher GFR [33]. Unfortunately, smoking status was rarely updated in the medical records, and we could only report it at baseline. We are unaware of any studies on how smoking habits change when mood stabilizing therapy is switched. We, therefore, had to assume that participants smoked as much on lithium therapy as on alternative mood stabilizers. In our study, smoking did not seem to affect eGFR slopes (Supporting Information Table A5).

In older studies, a ‘point of no return’ at a GFR of approximately 40 mL/min/1.73 m^2^ has been discussed, at which progression to kidney failure irreversibly continues despite lithium discontinuation. In this scenario, the underlying pathological process of lithium nephropathy (i.e., renal fibrosis) would become self‐perpetuating so that it could not be halted by lithium discontinuation [18]. Alternatively, lithium could have been discontinued too late, so that substantial tissue destruction already had taken place [34]. In our study, the positive effect of lithium discontinuation was most pronounced in participants with low eGFR. In most participants, this was a relative improvement. They continued to decrease in kidney function despite lithium discontinuation, albeit at a slower rate. In some, eGFR increased, a sign of renal recovery. Recovery of kidney function even occurred in participants with eGFR <30 at the time of lithium discontinuation. Participants who showed signs of recovery did not have higher eGFR than those who did not. This finding is in conflict with Hoekstra et al. [13]. In this study, 13 patients discontinued lithium with an eGFR <60. Of these, six patients continued to decline in kidney function. They had lower eGFR than patients with eGFR improvement (32 vs. 46 mL/min/1.73 m^2^). It is possible that these patients had other causes of low eGFR than lithium nephropathy. In our study, we censored measurements in the setting of severe illness or AKI and excluded glomerular diseases with rapid GFR decline. We also corrected for diabetes mellitus and hypertension. The kidney damage caused by lithium is highly variable between individuals [3]. For unknown reasons, some seem more susceptible than others. Participants with low eGFR tended to have a steeper decline of kidney function before discontinuation (Supporting Information Table A4). If this decline was due to lithium nephropathy, it would make sense that this group had the highest benefit of its discontinuation. We did not investigate the exact cause of low eGFR but in our previous study examining kidney function in the LiSIE cohort, lithium nephropathy was the most common cause in lithium‐treated participants [3].

Our study had several strengths. First, we were able to include a relatively large number of participants with proven lithium exposure of at least 4.5 years with a mean exposure of over 15 years. Second, we were able to meticulously validate our data with medical records going back as far as the 1960s. Therefore, contrary to registry studies, we could accurately determine lithium treatment times and check for other causes of increased creatinine. Third, the mirror‐image design approximated to a natural experiment. By letting participants act as their own controls, we were able to adjust for potential confounders. Studying annual change of eGFR in terms of slopes can also have advantages over absolute eGFR change as an outcome, allowing for sufficient power in smaller populations with shorter follow‐up.

Our study also had limitations. The study was retrospective and observational in nature. Reliance on medical records meant that the quality of our data was determined by the quality of the documentation. Further, this study was conducted in a single geographical area in an ethnically relatively homogenous population and not designed to specifically address sex‐differences. We possibly had a selection bias as participants discontinuing lithium might have had a steeper GFR slope than participants continuing lithium. This could have led to an overestimation of the magnitude of eGFR loss during lithium treatment.

In 25% of participants, lithium was stopped due to low eGFR. Serum‐creatinine tends to fluctuate over time, basically because of changes in hydration and muscle activity. If the decision to discontinue lithium was based on a temporarily low eGFR, this would have created a ‘regression toward the mean’ effect. We find this unlikely to be a major source of bias: Our mixed model analysis reflects up to 5 years of data with several measurements before and after lithium discontinuation. This reduces the effects of single low eGFR measurements on the slope. A ‘regression toward the mean’ effect would occur directly after lithium discontinuation. In our study, the effect on the mean annual eGFR was preserved over the complete post‐mirror period (Fig. 2). Finally, the annual loss of eGFR reverted to pre‐discontinuation levels after restarting lithium. This implies a causal effect of lithium.

We could not fully account for the differences in the treatment of CKD that took place during the study period. Sodium‐glucose transporter protein 2 inhibitors had not been generally adopted or promoted during the observation period. Around 35% of the participants had CKD stage G3b or worse. In these, it can be expected that improvements were made in the treatment of hypertension and diabetes. This includes a widespread adoption of RAAS‐inhibitors. There was indeed a higher prescription rate of RAAS‐inhibitors in the post‐mirror period. This might have led to an underestimation of the benefit of discontinuation in participants with CKD in the short run and/or to an overestimation of the effect in the long run. Yet, the absolute difference in RAAS‐inhibition was small, and we believe that this only had marginal effect.

Finally, there was a loss of participants for follow‐up in the post‐mirror period. This was mainly due to lithium having been reinstated after a short lithium‐free period or lithium having been discontinued because of severe physical illness. In participants discontinuing lithium because of severe illness, creatinine‐based eGFR could have either declined more rapidly due to comorbidities or more slowly due to weight and muscle loss. To avoid this, we excluded measurements that coincided with significant weight loss. In this respect, measured GFR or cystatin‐C‐based eGFR would have been better but was available in only few participants.

Importantly, potential renal benefits of lithium discontinuation need to be weighed against potential risks to mental health. The risk of relapse into an affective episode can be substantial [28], and the overall available evidence provides strong support for a suicide‐protective effect of lithium [35, 36]. In the LiSIE cohort, patients with BD‐1 or SZD had a fourfold increase in mean number of admissions and a threefold increase in mean number of bed days in the 2 years following lithium discontinuation [37]. Interestingly, there was no change in patients with BD‐2 or unspecified BD. For the whole sample, there was no significant change in suicidal and/or non‐suicidal self‐injuries. However, for a subgroup of patients, an increase in such events was seen. Suicide is a rare event, and the study was underpowered in that respect. In the current study, the renal effect of lithium discontinuation did not differ between BD‐1 and SZD or BD‐2 and unspecified BD (Supporting Information Table A5).

## Conclusions

Our results suggest a more favourable renal prognosis after lithium discontinuation, even at low eGFR. The beneficial effect of lithium discontinuation seems to persist over at least 5 years. In terms of long‐term kidney outcome, earlier rather than later lithium discontinuation will probably be more favourable. Once lithium is restarted, the eGFR decline can be expected to resume to previous levels. We need to better understand which factors put lithium‐treated patients on steep trajectories of eGFR decline and how best to counter such factors. Equally, we need to better understand which patients can discontinue lithium safely in terms of mental health and which cannot. The potential for an improved kidney prognosis must be weighed against the potential for a worse psychiatric course with an increased risk of relapse into an affective episode and suicide. A patient‐centred, not a kidney‐centred approach is needed to find the best balance between mental and renal health.

## Author contributions

Ursula Werneke and Michael Ott conceived the LiSIE study. Filip Fransson, Ursula Werneke, P. Andreas Jonsson and Michael Ott contributed to the conceptualization of the current study. Filip Fransson, Louise Öhlund and Michael Ott had access to the LiSIE raw data and validated the data. Filip Fransson with assistance of Per Liv, statistician (see acknowledgement), conducted the main statistical analysis. Filip Fransson and P. Andreas Jonsson worked on interpretation of historical creatinine measurements.  Filip Fransson and Michael Ott produced the initial draft of the manuscript. Filip Fransson, Ursula Werneke, Louise Öhlund, P. Andreas Jonsson and Michael Ott contributed to manuscript review and editing. Filip Fransson and Michael Ott contributed to data visualization. Filip Fransson and Michael Ott have accessed and verified all data. No author was precluded from accessing data in the study. All authors have read the final version of the manuscript and accept responsibility to submit for publication.

## Conflicts of interest statement

U.W. has received lecture honoraria from Lundbeck and Janssen and has served/serves on scientific committees for Janssen and Teva, receiving honoraria for these activities. M.O. had been scientific advisory board member for AstraZeneca AB, Sweden 2018–2020. F.F., L.Ö. and P.A.J. declare no conflicts of interest.

## Supporting information




**Table A1**: Published studies on change of glomerular filtration rate after lithium discontinuation.
Literature review

## Data Availability

The LiSIE datasets generated and analysed for the current study are not publicly available because of a lack of ethics committee permission and not having been part of the consent process. The structure of the dataset and the coding specification are available from the authors. Any other reasonable request will be raised with the Swedish Ethical Review Authority and health‐care provider.
