# Supplementary material for: Kidney function decline improves after lithium discontinuation
Source: J Intern Med. 2025 Jan 20;297(3):289–99. doi: 10.1111/joim.20054 (PMC11846072; doi:10.1111/joim.20054)
Supplement: Supplementary file 1 — Table A1: Published studies on change of glomerular filtration rate after lithium discontinuation. Literature review [file JOIM-297-289-s001.docx]

**Appendix**

**Table A1:** Published studies on change of glomerular filtration rate after lithium discontinuation

| First author | Year | Event investigated | Kidney function measured as | Groups | N | Time from event to discontinuation [years] | Time in study before discontinuation [years] | Kidney function at event studied, mean, (SD if reported) [ml/min/1.73m^2^] | Kidney function at end of follow-up, mean (SD if reported) [ml/min/1.73m^2^] | Annual change of kidney function before event, mean, (SD if reported) [ml/min/1.73m^2^] | Annual change of kidney function after event, mean, (SD if reported) [ml/min/1.73m^2^] | Follow-up [years] | Number of patients at last follow-up | Effect of somatic co-morbidities on kidney function investigated | Outcome kidney function after lithium discontinuation |
| --- | --- | --- | --- | --- | --- | --- | --- | --- | --- | --- | --- | --- | --- | --- | --- |
| **Prospective studies** | | | | | | | | | | | | | | | |
| Bendz | 1996 | Discontinuation | mGFR | Discontinuers | 13 | 0 | NA | 69 (SD 14.4) | 74 (SD 14.4) | NR | NR | 8.5 weeks | 13 | No | NS |
| Bendz | 1985 | Discontinuation | CrCl  mGFR | Discontinuers | 46 | 0 | NA | CrCl: 93.3  mGFR: 79.2 | CrCl: 96.5  mGFR: 88.2 | NR | NR | Mean 3 months | 42 (CrCl) 19 (mGFR) | No | CrCl: NS  mGFR: Improved* |
| **Retrospective studies** | | | | | | | | | | | | | | | |
| Hoekstra | 2022 | Discontinuation | eGFR | Discontinuers | 43 | 0 | Min 0.5 | NR | NA | NR | +2.0 relative to before discontinuation | Min 0.5 | NA | No | Improved** |
| Pahwa^a^ | 2021 | CKD <60 | eGFR | Discontinuers | 19 | Mean 0.5 (SD 0.7) Median 0.2 (IQR 0–0.9) | NA | 45.33 (SD 8.94) | 44.06 (SD 11.50) | NR | NR | Mean 4.9 (SD 5.3) | NA | Hypertension, diabetes mellitus | NS difference compared to continuers |
|  |  |  |  | Continuers | 20 | NA | NA | 50.16 (SD 7.85) | 48.57 (SD 8.74) | NR | NR | Mean 3.5 (SD 4.4) | NA | Hypertension, diabetes mellitus |  |
| Bocchetta | 2015 | CKD <45 | eGFR | Discontinuers + low-dose (<0.5 mmol/L) | 45 | NR | NA | 37.6 | 32.1 | NR | NR | 4 | 15 | No | NS difference compared to continuers^b^ |
|  |  |  |  | Continuers | 54 | NA | NA | 39.3 | 36.2 | NR | NR | 4 | 24 | No |  |
| Rej | 2013 | CKD <60 | eGFR | Discontinuers | 10 | Mean 0.82 | NA | 49.8 | 51.1 | NR | NR | 5 | 3 | Hypertension, diabetes mellitus | NS |
|  |  |  |  | Continuers | 17 | NA |  | 50.7 | 41.9 | NR | NR | 5 | 11 | Hypertension, diabetes mellitus | NS |
| Janowsky | 2009 | Peak creatinine in study | eCrCl | Discontinuers | 39 | NR | NR | 70.5 | 76.8 | NR | NR | NR | 39 | No | Improved* |
|  |  |  |  | Continuers | 18 | NA |  | 78.3 | 86.9 | NR | NR | NR | 18 | No | NS |
| Presne | 2003 | Discontinuation | eCrCl | Discontinuers | 11 | 0 | Min 1 | NR | NR | NR | NR | Min 1 | 11 | No | NS |
| Hetmar | 1991 | 10 years since last examination | mGFR  CrCL | Discontinuers | 8 | NR | NR | mGFR: Median 90.0 (IQR 22.8)  CrCL: Median 86.4 (IQR 12) | mGFR: Median 75 (IQR 17.4)  CrCL: Median 75.6 (IQR 30) | NR | NA | 10 | 8 | No | NS difference compared to continuers |
|  |  |  |  | Continuers | 19 | NA | NA | mGFR: Median 82.8 (IQR 19.2)  CrCL: Median 86.4 (IQR 19.2) | mGFR: Median 75 (IQR 34.8)  CrCL: Median 70.2 (IQR 18.6) | NR | NA | 10 | 19 | No |  |
| Vestergaard | 1981 | 20 months since last examination | CrCl | Discontinuers | 37 | Mean -14.4 (7.9) months | ~6 months | Only graphically | 90.6 | NR | NA | 20 months | 37 | No | NS difference compared to continuers |
|  |  |  |  | Continuers | 118 | NA | NA | Only graphically | 89.4 | NR | NA | 20 months | 118 | No |  |
| **Current study** | | | | | | | | | | | | | | | |
| Fransson | Current study | Discontinuation | eGFR | Discontinuers | 168 | 0 | 5 | 67.39 (SD 22.92) | NA | -1.58 | -0.02 | Max 5.0  Mean 2.98 (SD 2.13) | 139 | Hypertension, diabetes mellitus | improved*** |

N = number of individuals studied; mGFR = measured GFR by [^51^Cr] EDTA; CrCl = 24h-endogenous creatinine clearance; eGFR = estimated glomerular filtration rate; eCrCl = estimated creatinine clearance; NR = not reported; NA = not applicable; NS = non-significant; * p<0.05; ** p<0.01; *** p<0.001, ^a^Also see Kumar et al. 2023 for additional data using partially identical data set, ^b^p=0.01 in favour of continuation at re-analysis, see Bocchetta et al. 2023

**Literature review**

We searched PubMed for studies published between Jan 1 2003 and Aug 11 2024 using the search string “lithium AND kidney” or “lithium AND renal”, without language restrictions. We also searched PubMed for systematic reviews using the same search strings without time-restriction. We identified 1271 studies and 31 systematic reviews. These, we screened for data on the effect of lithium discontinuation on kidney function, reported either as glomerular filtration rate (GFR), or creatinine clearance. We then complemented the search with additional references from the identified studies and systematic reviews.

**METHOD**

**Participants**

**Kidney diseases**

Patient records were reviewed for incidence of kidney diseases. Patients with a confirmed diagnosis (i.e., kidney biopsy verified or strong clinical evidence supporting a specific diagnosis without biopsy) expected to have affected the kidney function with an irregular or rapidly declining eGFR pattern in the pre-mirror, post-mirror or after restarting lithium period were excluded. This resulted in the exclusion of one patient with rapidly progressing glomerulonephritis (RPGN) of unknown cause and of one patient with RPGN due to membranoproliferative glomerulonephritis. Patients with suspected or biopsy-verified nephrosclerosis, diabetic nephropathy, treated postrenal disease, stable chronic glomerulonephritis (IgA nephropathy, one patient) or lithium nephropathy were not excluded.

**Psychiatric diagnosis**

The diagnoses of bipolar disorder or schizoaffective disorder were assigned when a patient had received a diagnosis of either condition on at least two occasions at least 180 days apart. We also assigned a bipolar disorder diagnosis when patients had at least one manic and one depressive episode. We excluded patients in whom, after manual validation, a diagnosis of schizophrenia or personality disorder was more likely than that of bipolar disorder. The diagnosis at lithium insertion was established by reading and by manually reviewing the medical records until date of lithium insertion. Records from 1965 until Dec 31st Dec 2015, were included. During the time period reviewed, clinicians had diagnosed according to DSM or ICD in their various editions. To establish the BD diagnosis and subtype at the point of lithium insertion, we created a summary diagnosis as an approximation according to DSM-5. (Öhlund et al. 2019)

**Definitions of co-morbidities**

**Arterial hypertension**

Hypertension was set from the first time the diagnosis was set (ICD codes I10, I12, I13, I15) or the first prescription of anti-hypertensive medication, whichever came first. For anti-hypertensive medications, ATC-codes C02, C03, C07, C08 and C09 were used. ATC-code C03 or C07 were solely regarded as anti-hypertensive medication if the indication specified in the prescribing information for the patient or in the case records was blood-pressure lowering.

**Diabetes mellitus**

Diabetes mellitus was set if a diabetes diagnosis (ICD codes E10-14) was present. Alternatively, when a patient was treated with anti-diabetic medication (ATC-code A10). If a patient had anti-diabetic medication prescribed without a documented diagnosis of diabetes, the diagnosis was validated in the case records. Some patients had been prescribed anti-diabetics for other causes, e.g. metformin for polycystic ovary syndrome (PCOS). For the time of onset of diabetes, we used the first pathological HBA1c (>6.5% or >48 mmol/mol), the first prescription, or the first diagnosis, whichever was first. If the patient only had pathological HbA1c but neither diagnosis of diabetes mellitus nor anti-diabetic treatment, the patient was not included as having diabetes.

**Smoking**

Smoking was defined as reported current smoking at any time as recorded in the electronical medical records from Jan 1 1997 to Dec 31 2017. Non-smoking was defined as either reported non-smoking (and no future mention of current smoking) or past smoking before Jan 1 1997

**Cardiovascular disease**

Medical records were screened for ICD-codes I20-I25, I50, I110, I130, I132, I63-I66, I69, I739, I74, G45-G46. The starting date was set to the first mention of any of these events in the case records.

**Renin-angiotensin-aldosterone-inhibitors**

Any prescription of either ATC-code C09 or C03DA, the exposure-time was estimated from the prescription amount and prescribed dosage.

**Mirror periods**

**Figure A1** Study design


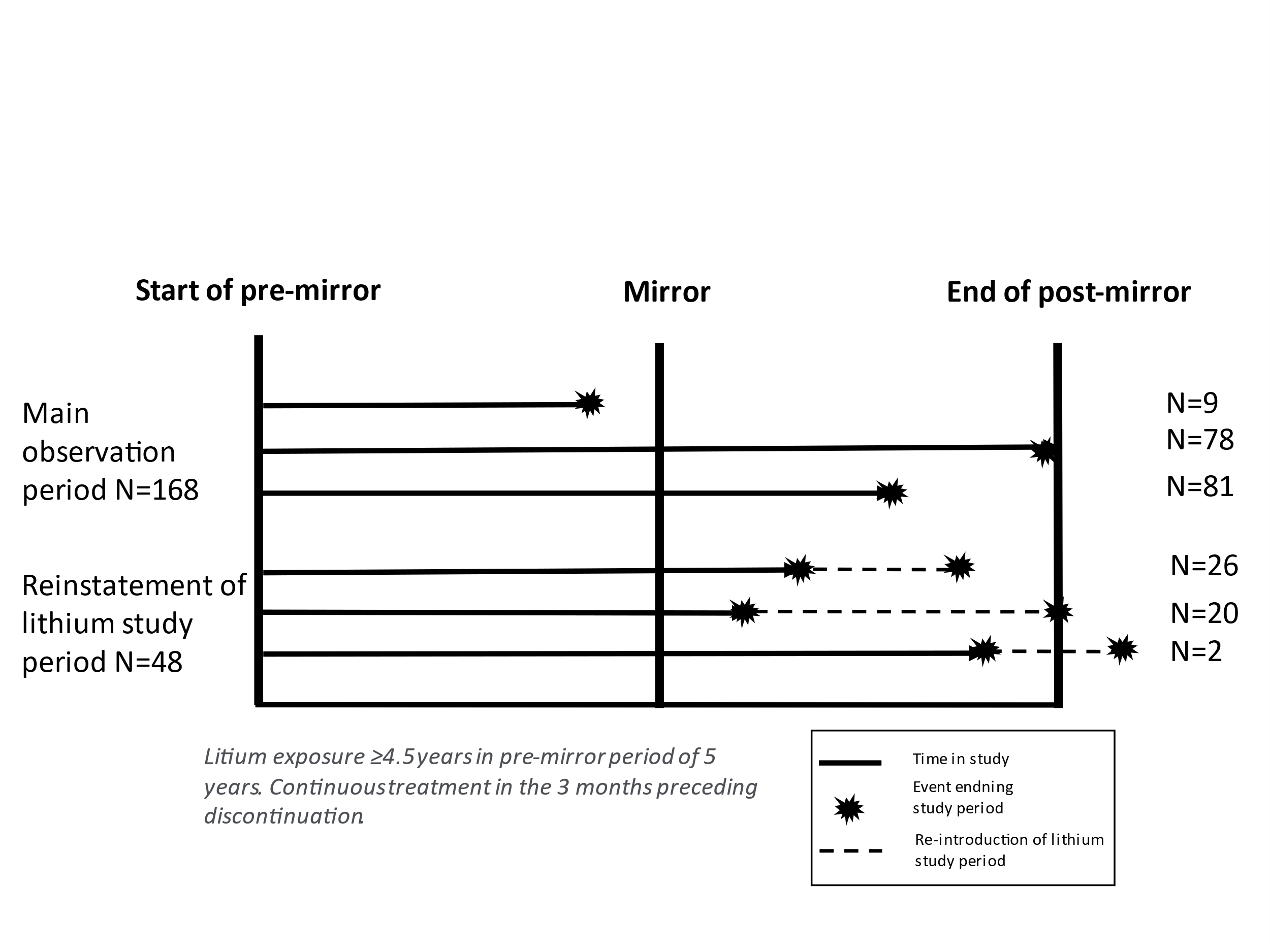


**Lithium dosage and type before discontinuation (pre-mirror period)**

146 participants were prescribed lithium sulphate exclusively. 19 Participants were switched from lithium citrate to lithium sulphate. 3 participants were prescribed lithium citrate exclusively.

146 participants were prescribed lithium twice daily. 14 participants were prescribed once daily. Four participants were prescribed every other day, two were prescribed an unknown dosage and one was prescribed thrice daily.

Usually, lithium concentration measurements were scheduled 12 hours after ingestion.

**PROCEDURES**

**Measurement of kidney function and estimation of GFR**

**Censoring for acute kidney injury (AKI)**

Individual patient’s creatinine measurements were screened. If the difference between two consecutive measurements was 27 µmol/L or more (AKI grade 1, KDIGO 2012), the case records were consulted for possible causes of AKI. If a probable cause was found, the creatinine measurements were censored until (a) creatinine had returned to baseline (<27 µmol/L difference), or (b) three months had passed (now being regarded as chronic kidney disease). Additionally, to avoid bias from medical treatment and procedures, creatinine measurement after AKI were censored during the inpatient stays.

**STATISTICAL ANALYSIS**

**Mixed model design**

The outcome was the difference of the annual change of eGFR between the pre- and post-mirror periods calculated using the mixed effects model. Fixed effects were age, sex, “years since discontinuation of lithium”, hypertension, and diabetes. All effects on the slope were assumed to be linear. The patients’ intercepts and slopes were included as mutually correlated random effects. The continuous time-dependent variable “years since discontinuation of lithium”, represented the change in slope after discontinuation had occurred. Hypertension and diabetes were included as continuous time-dependent variables starting at the time of diagnosis or at the start of the pre-mirror period if the patient had already been diagnosed before the study. Sex was included as a dichotomous variable.

The annual eGFR change in the pre-mirror period was the effect of age on eGFR after adjusting for sex, hypertension, and diabetes. The annual eGFR change in the post-mirror period was the effect of age on eGFR after adjusting for sex, hypertension and diabetes combined with the effect of discontinuing lithium.

To represent the effect on slope of re-instating lithium the additional fixed effect ‘years since re-instating lithium’ was included.

**Syntax**

Mixed effects model, main model

MIXED eGFRLMrev By Sex WITH Age Litihumfreetimeyears Restartinglithiumtimeyears Diabetestimeyears Hypertensiontimeyears

/FIXED Age Sex Lithiumfreetimeyears Restartinglithiumtimeyears Diabetestimeyears Hypertensiontimeyears | SSTYPE(3)

/RANDOM INTERCEPT Age | SUBJECT(PatientID) COVTYPE(UN)

/PRINT SOLUTION TESTCOV.

Mixed effects model stratified for different ranges of eGFR at discontinuation.

MIXED eGFRLMrev By Sex Discontinuationgroup WITH Age Lithiumfreetimeyears Diabetestimeyears Hypertensiontimeyears

/FIXED Age Sex Lithiumfreetimeyears Diabetestimeyears Hypertensiontimeyears Discontinuationgroup* Lithiumfreetimeyears | SSTYPE(3)

/RANDOM INTERCEPT Age | SUBJECT(PatientID) COVTYPE(UN)

/PRINT SOLUTION TESTCOV.

**RESULTS**

**Severe disease or medical procedures ending post-mirror period**

Some diseases or medical procedures severely affect serum-creatinine levels and therefore compromise creatinine-based GFR estimation. In these patients, only eGFR values before the time of diagnosis/event were included in the analysis. These reasons were: disseminated cancer with weight loss in ten patients, palliative care or major weight loss due to other causes (post-stroke, progressive dementia, post-pneumonia) in six patients, gastric bypass with weight loss in three patients.

**Table A2** Comparison of key parameters pre- vs. post-mirror

|  | **Pre-mirror period, lithium exposed**  **N=168** | **Post-mirror period, lithium discontinued**  **N=159^a^** | **p-value** |
| --- | --- | --- | --- |
| **Time in study, (years) Mean (SD)** | 4.96 (0.31) | 2.98 (2.13) |  |
| Minimum – maximum | 2.13-5.00 | 0.00-5.00 |  |
| Median (IQR) | 5.00 (0.00) | 4.15 (4.46) |  |
| Patient years | 832.87 | 501.74 |  |
| **Arterial hypertension N (%)** | 72 (42.86) | 79 (49.69) |  |
| **Diabetes mellitus N (%)** | 28 (16.67) | 32 (20.13) |  |
| **Participants with creatinine samples N (%)** | 166 (98.81) | 139 (87.42) |  |
| Number of samples per participant, mean (SD) | 22.40 (11.25) | 7.85 (8.46) |  |
| Minimum – maximum | 0-79 | 0-38 |  |
| Median (IQR) | 21 (12.00) | 5 (10.00) |  |
| Samples/year**^b^,** mean (SD) | 4.52 (2.24) | 2.49 (3.16) | <0.0001 |
| **RAAS-inhibitors^c^, N (%)** | 32 (19.05) | 34 (20.24) |  |
| Time on RAAS-inhibition/year mean in percent (SD) | 9 (24) | 13 (31) | 0.0092 |
| **Participants with AKI N (%)** | 31 | 17 |  |
| Episodes | 39 | 25 |  |
| Episodes/year^b^, mean (SD) | 0.047 (0.11) | 0.045 (0.16) | 0.95 |

N= number; IQR = interquartile range; SD = standard deviation, eGFR = estimated glomerular function, RAAS = renin-angiotensin-aldosterone-system, AKI = Acute kidney injury.
^a^Post-mirror N=159 due to nine participants having an event ending the study in the pre-mirror period due to severe disease or medical procedure.
^b^Minimum time set to one year.
^c^Renin-angiotensin-aldosterone-system-inhibitors, defined as any prescription of either ATC-code C09 or C03DA, the exposure-time was estimated from the prescription amount and prescribed dosage.

**Table A3** Event ending analysis after restarting lithium, N=48

|  | **N (%)** |
| --- | --- |
| End of mirror | 20 (41.67) |
| Discontinuing lithium again | 9 (18.75) |
| Death | 6 (12.50) |
| Severe disease or medical procedure | 6 (12.50) |
| End of LiSIE study period | 5 (10.42) |
| Mirror extended 2 years after restarting lithium | 2 (4.17) |
| Chronic renal replacement therapy | 0 (0) |

**Figure A2** Mean annual eGFR stratified by eGFR at discontinuation.


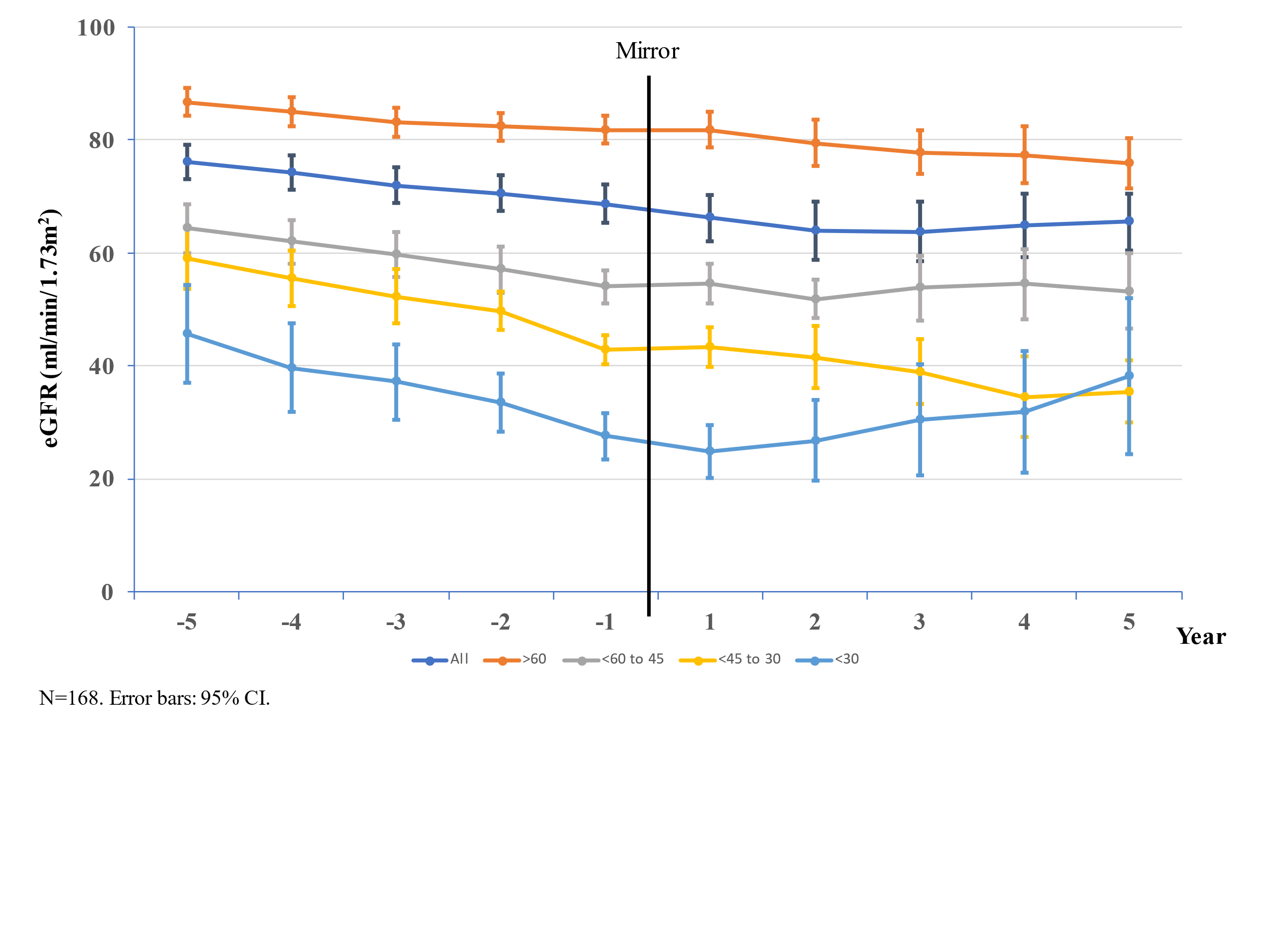


**Table of data in Figure A2**

| Years: | -5 | -4 | -3 | -2 | -1 | 1 | 2 | 3 | 4 | 5 |
| --- | --- | --- | --- | --- | --- | --- | --- | --- | --- | --- |
| **eGFR >60** Mean (SD)  N | 87 (13) 97 | 85 (14) 104 | 83 (13) 104 | 82 (13) 104 | 82 (13) 106 | 82 (14) 73 | 79 (14) 45 | 78 (12) 37 | 77 (16) 41 | 76 (14) 41 |
| **All** Mean (SD)  N | 76 (19) 154 | 74 (20) 161 | 72 (20) 162 | 71 (21) 161 | 69 (22) 163 | 66 (23) 124 | 64 (23) 77 | 64 (21) 63 | 65 (23) 64 | 66 (20) 62 |
| **eGFR <60 to 45** Mean (SD)  N | 64 (11) 26 | 62 (10) 27 | 60 (11) 27 | 57 (11) 26 | 54 (8) 26 | 55 (9) 24 | 52 (6) 14 | 54 (10) 12 | 55 (10) 10 | 53 (11) 11 |
| **eGFR <45 to 30** Mean (SD)  N | 59 (12) 17 | 56 (11) 17 | 52 (10) 17 | 50 (7) 17 | 43 (5) 17 | 43 (7) 14 | 42 (8) 9 | 39 (8) 8 | 35 (10) 7 | 36 (7) 7 |
| **eGFR <30** Mean (SD)  N | 46 (16) 14 | 40 (14) 13 | 37 (13) 14 | 33 (10) 14 | 28 (8) 14 | 25 (9) 13 | 27 (11) 9 | 31 (12) 6 | 32 (13) 6 | 38 (12) 3 |

**Additional analysis using linear regression (least-squares)**

For this, we included all patients with at least three creatinine measurements over a period of at least one year before and after discontinuation, respectively. For patients restarting lithium treatment, we analysed the annual change of eGFR when three or more creatinine measurements were available over a period of at least 180 days after restarting lithium.

Annual change of eGFR could be calculated in 163 patients before discontinuation, in 92 patients after discontinuation and in 36 patients after restarting lithium.
Patients were stratified in sub-groups according to last eGFR before lithium discontinuation. Annual change of kidney function before and after discontinuation was compared using paired t-test.

**Table A4** Subgroup analysis, summary measure of slopes

| **Last eGFR before discontinuation (ml/min/1.73m^2^)** | **Pre-mirror (ml/min/1.73m^2^/year)** | **Post-mirror (ml/min/1.73m^2^/year)** | **Paired samples t-test** |
| --- | --- | --- | --- |
| All | -1.96 (SD 2.68, median -2.02 IQR 3.24) (N=163) | -1.30 (SD 3.99, median -0.95, IQR 2.94) (N=92) | p=0.06 (88 pairs) |
| <60 | -3.21 (SD 2.64, median -2.86, IQR 2.66) (N=57) | -0,74 (SD 2.69, median -0.97 IQR 3.01) (N=34) | p=0.0001 (33 pairs) |
| <45 | -4.06 (SD 2.68, median -3.79 IQR 2.65) (N=31) | -1.06 (SD 2.96, median -1.07 IQR 3.97) (N=20) | p=0.007 (19 pairs) |
| <30 | -4.25 (SD 2.91, median -3.44 IQR 2.55) (N=14) | -1.30 (SD 2.99, median -1.01 IQR 3.74) (N=10) | p=0.04 (10 pairs) |

After reinstating lithium, a summary measure of mean annual eGFR change could be calculated in 36 patients to -2.36 (SD 2.82) ml/min/1,73m^2^/year**.** Compared to before discontinuation there was no significant difference, paired samples t-test, 36 pairs, p= 0.42. Only eight patients had sufficient data on both, annual change of kidney function after discontinuation and after reinstating lithium treatment. In these patients, annual change was –3.05 (SD 1.63) after discontinuation and –3.73 (SD 4.77) after reinstating lithium. The difference was not significant (p=0.66, paired-samples t-test, two-tailed).

**Table A5** Comparison of baseline characteristic in patients in whom eGFR-decline was reverted or attenuated (N=53) compared to patients in whom it was not (N=35)

|  | **Chi-square test** |
| --- | --- |
| BD-1/SZD or BD-2/unspecified bipolar | p=0.96 |
| Sex | p=0.7 |
| Age ≥ 65 | p=0.34 |
| Current or former smoking | p=0.57 |
| Hypertension | p=0.07***** |
| Diabetes | p=0.058***** |
| Life-time lithium exposure ≥= 10 years | p=0.059***** |
| Reduced GFR as cause of discontinuation | p=0.017***** |
| Last eGFR before discontinuation <60 | p=0.0059***** |
| Annual eGFR decline >2 ml/min/1.73m^2^/year | p<0.0001***** |

*Higher proportion of reverted or attenuated eGFR decline in patients with hypertension, diabetes, life-time lithium exposure ≥ 10 years, reduced GFR as cause of discontinuation, last eGFR before discontinuation <60, annual eGFR decline >2 ml/min/1.73m^2^/year.

**Table A6** Proportion of patients with improved slope of annual change of kidney function after lithium discontinuation (N=88)

| Annual change, ml/min/1.73m^2^/year | N | Improved slope^a^(N) | Positive slope^b^ (N) | eGFR at discontinuation, ml/min/1.73m^2^ (SD) |
| --- | --- | --- | --- | --- |
| All | 88 | 53 (60%) | 22 (25%) | 64.94 (23.29) |
| ≥0.0 | 16 | 2 (13%) | 3 (19%) | 82.69 (20.19) |
| <0.0 to-2.0 | 25 | 9 (36%) | 5 (20%) | 70.10 (21.21) |
| <-2.0 to -4.0 | 28 | 24 (86%) | 8 (29%) | 61.84 (20.74) |
| <-4.0 | 19 | 18 (95%) | 6 (32%) | 47.75 (19.85) |

^a^in these participants *annual change of kidney function* improved; ^b^in these participants *absolute kidney function* improved after discontinuation (recovery).
